# The Short Foot Plugger

**Published:** 1907-10

**Authors:** W. E. Driscoll

**Affiliations:** Braidenton, Fla.


### THE SHORT FOOT PLUGGER.

By W. E. Driscoll, D. D. S., Braidenton, Fla.

This looks like a diminutive subject for a formal paper claiming this society's attention. Still when I think of all that might with propriety be said in this connection, I am not sure where to begin, what to leave out, or where to stop. The man who habitually slights things because they are small has no place in dental practice. Dentistry is made up of "nice points."

"Small plugger points" might be a title as appropriate, did I not wish to focus attention on a plugger and its uses that I fail to find illustrated in any catalogue of instruments. The one so illustrated that is most readily changed to what I wish to describe is the Varney No. 5, illustrated on page 39 of the S. S. White Dental Mfg. Co.'s catalogue of Operating Instruments, of March 1906.

The change I make is to grind from the shank at the instep with a stump wheel to about two-thirds of its regular size, taking away also one-third of the serrated face. Right here I wish to say, I have been reducing the size of the pluggers I use for cohesive gold foil gradually for over thirty-five years. The reasons for so doing are several, and important. First, better anchorage, and cohesion. Second, less shock to patient in malleting and danger to periosteum, a most vital consideration in some cases.

Third, better adaptation of gold to cavity walls, and margins. Fourth, less danger of damaging the margin by striking it with too large an instrument. Fifth, easier access to many approximal cases.

With the Varney No. 5, changed as described, one is better equipped to carry cohesive gold along cervical walls to the margin in approximal cavities where condensation is necessarily directed toward such wall, rather than a sliding impact, toward bottom of cavity parallel with the cavity wall, for several reasons: First, we want a point so small that we may be sure the gold is carried solidly to that wall without any danger of bridging over slightly condensed parts. Second, the slant of the serrated face is at an angle with the body of the plugger that enables the operator to make this even condensation with more accuracy than with any other form. The nearest substitute in the Varney list is No. 3. The objections to this pattern for purpose just named is that it is almost sure to injure the margin, and a plugger with a larger serrated face of this class does not have the angle of No. 5 and for that reason is comparatively impracticable for accurate condensation of cohesive gold at a point such as is under consideration. This last mentioned No. 3 has its place, but it is not at this stage of the work. The foot long plugger is indispensable in its field also. There are other conditions in which this modified Varney No. 5 will do safer, and more accurate work than any other pattern. To carry conviction on this point you must have the instrument and try it.

It is for comparatively inaccessible approximal cavities where condensation must be made directly toward the cervical wall and a few other occasions, but occasions where the value of the operation turns upon the weakest point in the filling being made equal to the rest.

If any one thinks this is placing too much stress on a small part of the process of filling one class of cavities, I wish to say this: Is it not high time that some remedy is found for the decay around fillings we see within a year after they are inserted? Can there be doubt that in many cases moisture penetrates many of these cavities almost as soon as the rubber dam is removed? And does not every one know that it requires the utmost care in intricate places to prevent the plugger from damaging the cavity margin, leaving it ready for early resumption of decay? And can there be any question that part of this trouble comes from instruments not best suited for such part of the work? With this modified Varney No. 5 in hand, the careful operator will realize after a trial that he has something indispensable for accurate work in positions I have described, and no doubt will wonder why an instrument of such importance has not long ago been illustrated in catalogues and kept on sale.

I will admit that for non-cohesive gold foil comparatively larger points are required for at least part of the work. But we see many cases where we are not justified in so deepening shallow cavities so they will retain non-cohesive gold merely to gain any advantage there may be in this style of gold. This is especially true in very sensitive dentine. Larger points are also required in using crystal and moss-fibre cohesive gold, and to some degree for every thin cohesive foil. Yet I am sure a very large percentage of leaky fillings of the past and present are due to plugger points used being to large, and of improper forms for certain conditions. Would it not be well if we should have more individuality in the choice of instruments we use? When one feels the need of an instument not illustrated in the catalogues, why not select the one most readily changed to what is wanted, and make the change himself, or have the instrument maker carry out his ideas? This is the way progress was made in the past, and I do not believe that improvement in our professional appliances will stop where we now are. So then let each one help here as well as elsewhere, for improvement and progress in our methods.

